# Towards real-time cardiovascular magnetic resonance-guided transarterial aortic valve implantation: In vitro evaluation and modification of existing devices

**DOI:** 10.1186/1532-429X-12-58

**Published:** 2010-10-13

**Authors:** Philipp Kahlert, Holger Eggebrecht, Björn Plicht, Oliver Kraff, Ian McDougall, Brad Decker, Raimund Erbel, Mark E Ladd, Harald H Quick

**Affiliations:** 1 Department of Cardiology, West-German Heart Center Essen, University Hospital Essen, University Duisburg-Essen, Hufelandstrasse 55, 45122 Essen, Germany; 2 Department of Diagnostic and Interventional Radiology and Neuroradiology, University Hospital Essen, University of Duisburg-Essen, Hufelandstrasse 55, 45122 Essen, Germany; 3 Evasc Medical Systems, 107-1099 West 8th Avenue, Vancouver, BC V6H 1C3, Canada; 4 Institute of Medical Physics, Friedrich-Alexander University Erlangen-Nürnberg, Henkestrasse 91, 91052 Erlangen, Germany

## Abstract

**Background:**

Cardiovascular magnetic resonance (CMR) is considered an attractive alternative for guiding transarterial aortic valve implantation (TAVI) featuring unlimited scan plane orientation and unsurpassed soft-tissue contrast with simultaneous device visualization. We sought to evaluate the CMR characteristics of both currently commercially available transcatheter heart valves (Edwards SAPIEN™, Medtronic CoreValve^®^) including their dedicated delivery devices and of a custom-built, CMR-compatible delivery device for the Medtronic CoreValve^® ^prosthesis as an initial step towards real-time CMR-guided TAVI.

**Methods:**

The devices were systematically examined in phantom models on a 1.5-Tesla scanner using high-resolution T_1_-weighted 3D FLASH, real-time TrueFISP and flow-sensitive phase-contrast sequences. Images were analyzed for device visualization quality, device-related susceptibility artifacts, and radiofrequency signal shielding.

**Results:**

CMR revealed major susceptibility artifacts for the two commercial delivery devices caused by considerable metal braiding and precluding in vivo application. The stainless steel-based Edwards SAPIEN™ prosthesis was also regarded not suitable for CMR-guided TAVI due to susceptibility artifacts exceeding the valve's dimensions and hindering an exact placement. In contrast, the nitinol-based Medtronic CoreValve^® ^prosthesis was excellently visualized with delineation even of small details and, thus, regarded suitable for CMR-guided TAVI, particularly since reengineering of its delivery device toward CMR-compatibility resulted in artifact elimination and excellent visualization during catheter movement and valve deployment on real-time TrueFISP imaging. Reliable flow measurements could be performed for both stent-valves after deployment using phase-contrast sequences.

**Conclusions:**

The present study shows that the Medtronic CoreValve^® ^prosthesis is potentially suited for real-time CMR-guided placement in vivo after suggested design modifications of the delivery system.

## Background

Transcatheter, transarterial aortic valve implantation (TAVI) is rapidly emerging as a promising new treatment option for patients with severe symptomatic aortic valve stenosis who are considered at high or prohibitive surgical risk[1,2]. Safe navigation of the valve delivery devices through the vasculature and precise placement of the stent-valve prostheses within the native aortic valve annulus are the challenging key steps of the procedure, which is currently performed under X-ray fluoroscopic and angiographic guidance. X-ray fluoroscopy and angiography, however, entail several shortcomings beyond the inherent harmful effects of radiation exposure which may be overcome by real-time cardiovascular magnetic resonance (CMR) guidance, offering real-time image acquisition with unrestricted scan plane orientation and superior, detailed soft-tissue contrast with simultaneous visualization of the interventional device[3,4] as demonstrated for a variety of vascular procedures in animal studies, including percutaneous transluminal angioplasties[5], selective embolization procedures[6], placement of atrial septal closure devices[7], peripheral[8,9] as well as coronary stent placement[10], stenting of aortic coarctation[11], and aortic stent-graft placement[12].

In the setting of TAVI, CMR guidance offers an improved intraprocedural guidance for navigation of the large-diameter catheters through the vasculature. Moreover, CMR appears to be particularly advantageous over X-ray fluoroscopy and angiography during valve implantation since it permits precise, real-time orientation for axial positioning and deployment of the prosthesis without the need for contrast media, not even gadolinium. This may also help to reduce the total amount of nephrotoxic contrast media potentially causing acute kidney injury which has been shown to increase the risk of postoperative mortality in the elderly TAVI patient population[13]. Although transesoephageal echocardiography including real-time 3D imaging is used as a helpful adjunct imaging modality[14], this technique has not eliminated the need for fluoroscopy and angiography.

In addition to an improved procedural guidance, CMR may also be useful for preinterventional diagnostic evaluation of the aortic valve aortic valve[15,16] and the vascular aortic anatomy for interventional planning[17] as well as for immediate evaluation of the result[18-20]. Furthermore, TAVI appears particularly attractive for a CMR-guided approach, since device delivery is performed through large-diameter vessels, i.e. the femoral or subclavian artery, and the delivery devices come with a rather large instrument caliber of 18 to 24 French outer diameter. Hence, TAVI is amenable to passive catheter tracking and may be performed without the need for additional device modifications as needed for active CMR visualization approaches[3,4].

In the present study, the CMR characteristics of the two currently commercially available transcatheter heart valves with their dedicated transfemoral delivery devices (Edwards SAPIEN™, Edwards Lifesciences Inc., Irvine, CA, USA; Medtronic CoreValve^®^, Medtronic, Inc., Minneapolis, MN, USA) and of a custom-built delivery device for the Medtronic CoreValve^® ^bioprosthesis with design modifications targeted toward improved CMR safety and CMR compatibility have been systematically investigated in phantom experiments. This study is intended to serve as an initial step toward real-time CMR-guided TAVI in vivo.

## Methods

### TAVI Devices

**Figure 1:**
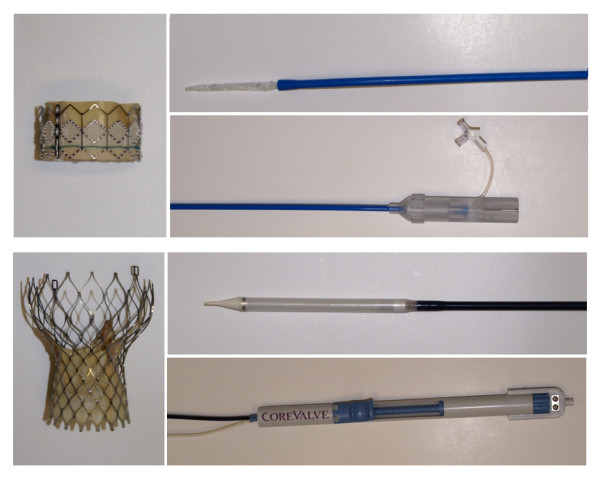
**TAVI Devices.** Photograph of the two commercially available devices for transarterial aortic valve implantation: the balloon-expandable, stainless steel-based Edwards SAPIEN™ stent-valve and its dedicated transfemoral RetroFlex delivery system (top) as well as the self-expandable, nitinol-based Medtronic CoreValve^® ^bioprosthesis and its original delivery catheter (bottom).

**Figure 2:**
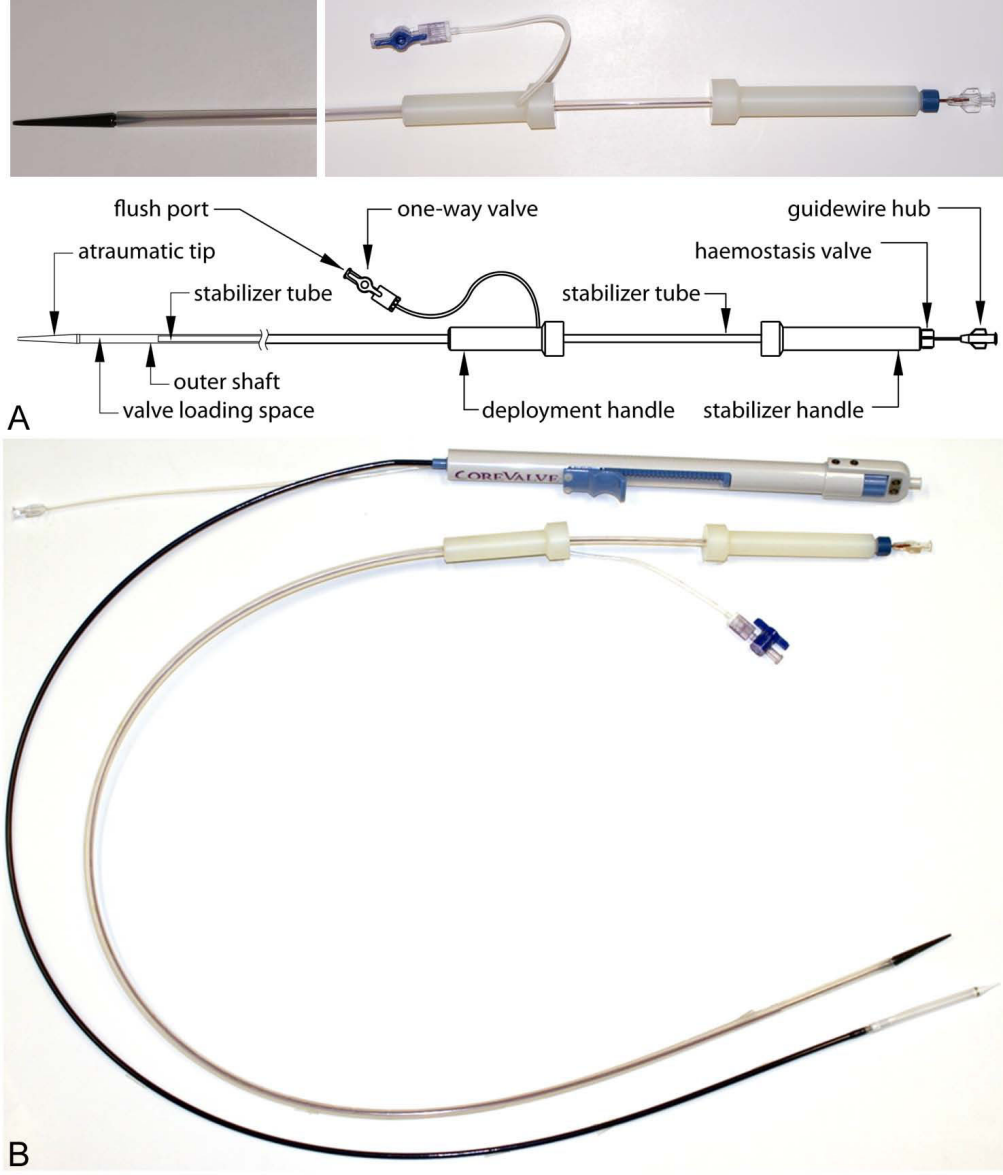
**Modified Delivery Device for the Medtronic CoreValve(R) prosthesis.** a. Photograph of the modified delivery device (top) and schematic drawing with labeling of the relevant parts (bottom). b. Photograph of the original, reinforced delivery device for the Medtronic CoreValve^® ^bioprosthesis and the custom-built, CMR-compatible device, displaying similar flexibility and rigidity under manually bending.

The two currently commercially available transcatheter heart valves - the balloon-expandable Edwards SAPIEN™ and the self-expandable Medtronic CoreValve^® ^bioprosthesis - and their dedicated transfemoral delivery devices were examined (Figure 1). Additionally, a custom-built, modified CMR-compatible delivery device for the Medtronic CoreValve^® ^bioprosthesis was designed and evaluated (Figure 2).

#### 1. Edwards SAPIEN™-Prosthesis and Delivery Device

The balloon-expandable Edwards SAPIEN™ prosthesis is a tubular, slotted, stainless steel stent with an attached uni-directional trileaflet bovine pericardial tissue valve and a fabric sealing cuff. Prior to implantation, the biosprosthesis is mounted and crimped onto a balloon catheter (Z-MED II, NuMED, Inc., Hopkinton, NY, USA) using a specially designed crimping device. When the stent-valve is correctly positioned within the native aortic valve annulus it is deployed by balloon inflation under partial cardiac arrest induced by rapid right ventricular pacing.

The dedicated delivery device of the Edwards SAPIEN™ prosthesis consists of a large-diameter over-the-wire guiding catheter of 22 or 24 French (for the 23 mm and the 26 mm stent-valve, respectively) which is used for advancing the prosthesis through the vasculature and tracking it through the aortic arch (RetroFlex, Edwards Lifesciences Inc., Irvine, CA, USA), and the aforementioned balloon catheter which protrudes distally from the guiding catheter. The guiding catheter shaft has a polytetrafluoroethylene-lined, stainless steel braided multidurometer PEBAX™/VESTAMID™ construction ensuring enough rigidity for pushing and torquing. An incorporated softer mid-distal section made of soft durometer PEBAX™ gives the catheter greater flexibility for propagation through the aortic arch and allows steerable deflection from 0 to 120 degrees. This is achieved by a laser-welded stainless steel pull wire which is connected to a slide nut and to guides that direct the pull wire and, thereby, the central lumen to the proper path when the rotational grip at the handle of the guiding catheter is operated (Figure 1).

#### 2. Medtronic CoreValve^® ^Bioprosthesis and Delivery Device

The self-expandable Medtronic CoreValve^® ^bioprosthesis is composed of a tri-level nitinol stent frame with an integrated uni-directional trileaflet porcine pericardial tissue valve. The inflow portion of the stent-valve has a high radial stiffness in order to anchor the stent within the calcified, native aortic valve leaflets; the middle portion is waisted, thereby maintaining coronary perfusion; and the outflow portion implants itself in the ascending aorta to orient the prosthesis parallel to the blood flow.

The delivery system is an over-the-wire catheter with a composite 12 French polytetrafluoroethylene, metal braided, Aesno nylon 12 shaft and an 18 French RILSAN^®^/PEBAX^® ^distal end ("capsule") sheathing the crimped prosthesis, which can be released stepwise with nearly continuous preservation of transaortic blood flow. Release of the prosthesis is achieved by turning a rear micro-knob at the polycarbonate, acrylonitrile-butadiene-styrene, acetal handle which causes antagonist movement of the sheath and an inner polyetheretherketone, stainless steel hypotube element, thereby progressively unsheathing and finally releasing the crimped stent-valve.

Engineering data and initial screening of both commercially available delivery systems (Edwards and Medtronic) for metallic components revealed reinforcement of the devices by ferromagnetic metal braiding, as well as use of ferromagnetic markers which may affect CMR compatibility and CMR safety. Both delivery devices thus revealed significant ferromagnetic attraction when brought near the magnet bore and showed ferromagnetic metal artifacts exceeding the device diameters. For this study, it was thus decided to develop and investigate a modified version of the delivery device for the Medtronic CoreValve^® ^bioprosthesis.

#### 3. Modified Delivery Device for the Medtronic CoreValve^® ^Bioprosthesis

Design modifications of the delivery device for the self-expandable, nitinol-based Medtronic CoreValve^® ^bioprosthesis towards improved CMR safety and compatibility was performed with the help of Evasc Medical Systems (Evasc Medical Systems, Vancouver, BC, Canada). For this purpose, the delivery catheter was designed completely without the use of metallic components such as marker bands or reinforcing wire braids. The modified device was constructed with a polytetrafluoroethylene outer shaft and a nylon stabilizer tube (Figure 2). The deployment of the stent-valve is achieved by holding the catheter's stabilizer handle in a fixed position while sliding the deployment handle, which is connected to the outer shaft, towards the stabilizer handle. The stabilizer maintains the position of the crimped stent valve while the outer shaft is retracted allowing stepwise expansion of the prosthesis in the desired location. This deployment method is common to many delivery catheters for self-expanding devices.

The design modification resulted in a slight but practically negligible increase in the crossing profile from 18 to 20 French - when compared to the original Medtronic CoreValve^® ^delivery catheter - in order to contain all relevant tubing and to match the relevant mechanical properties of the original, metal reinforced device, specifically flexibility and rigidity which are required for propagation of the device through the ilio-femoral vessels, the aorta and the aortic arch. Although flexibility and rigidity were not quantitatively bench-tested, the modified device showed similar properties to the original device when manually manipulated (Figure 2).

### In Vitro Phantom Setup

**Figure 3:**
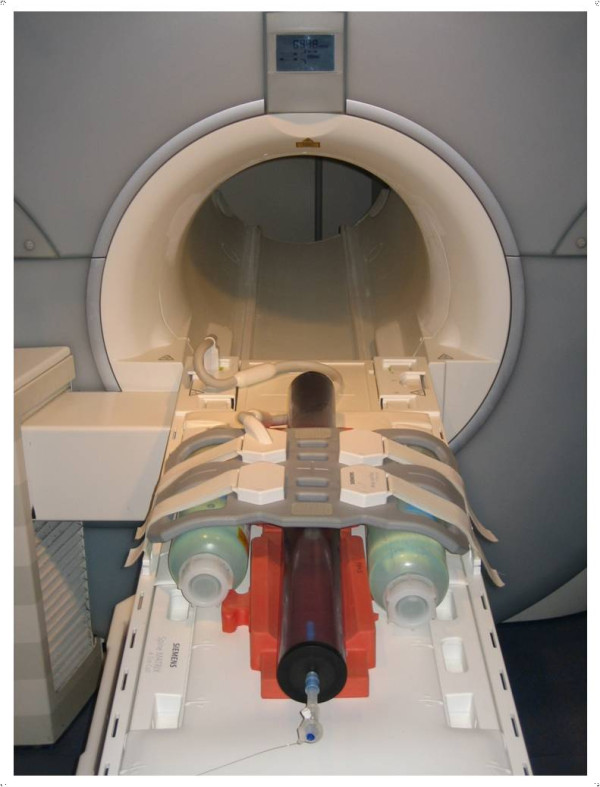
**Experimental setup.** The TAVI devices were contained in a Plexiglas phantom consisting of a 1-m-long, 10-cm-diameter tube that was placed longitudinal to the main magnetic field of the MRI scanner on top of a spine and below a body phased-array RF coil for signal reception. Four water-filled bottles were placed beside the phantom for RF transmit body coil loading.

Scanning was performed on a 1.5-Tesla whole-body CMR scanner (Magnetom Avanto, Siemens Healthcare Sector, Erlangen, Germany) equipped with gradients capable of a maximum amplitude of 40 mT/m and a slew rate of 200 T/m s^-1^. The stent-valves and their delivery systems were placed in a phantom containing water and gadolinium contrast agent (Omniscan, Amersham Health, Princeton, NJ, USA) at a concentration of 1:40. The phantom consisted of a 1-m-long, 10-cm-diameter Plexiglas tube with a central hole for linear catheter manipulation over a taut 0.8-mm-diameter Nylon cord simulating a guide-wire. At the proximal end, the phantom tube was equipped with a 30 French introducer sheath sealed by a silicone membrane to allow for introduction and rapid exchange of different vascular instruments. The phantom was designed to enable CMR assessment of the delivery device and the stent-valve artifact properties in static conditions as well as during manual catheter advancement and subsequent valve deployment utilizing real-time CMR. The phantom was placed longitudinal to the axis of the main magnetic field on top of a multichannel spine phased-array RF coil with three clusters of coil elements activated for signal reception (Tim Technology, Siemens Healthcare Sector, Erlangen, Germany). A two-element BodyFlex phased-array RF coil (Tim Technology, Siemens Healthcare Sector, Erlangen, Germany) was placed on top of the phantom for signal reception (Figure 3). This setting has been previously used by our group to evaluate thoracic aortic stent-grafts for real-time CMR-guided placement[21] prior to in vivo application[12].

### High-Resolution CMR

In an initial experiment, the expanded stent-valves and the delivery devices were separately imaged while suspended freely in the water bath phantom in order to determine device-related imaging artifacts. Imaging was performed using high-resolution T_1_-weighted 3D fast low-angle shot (FLASH) imaging to cover the whole extent of the devices (imaging parameters: repetition time (TR) 4.2 ms, echo time (TE) 1.7 ms, flip angle 40°, field-of-view (FOV) 220 × 90 mm^2^, matrix 512 × 204, 104 slices, bandwidth 510 Hz/pixel, acquisition time (TA) 3:09 minutes). The acquired spatial resolution resulted in 0.4 × 0.4 × 0.8 mm^3 ^voxel size.

### Real-time CMR

The delivery devices were subsequently examined under dynamic conditions with 2D real-time fast imaging with steady-state precession (rt-TrueFISP) sequences using a Cartesian reconstruction scheme (imaging parameters: TR 3.2 ms, TE 1.6 ms, flip angle 60°, FOV 300 × 150 mm^2^, matrix 256 × 128, bandwidth 1220 Hz/pixel, TA 0.41 seconds/image), both with and without the mounted stent-valves. The frequency-encoding direction was chosen parallel to the longitudinal direction of the devices and of the phantom; thus, it was also parallel to the direction of the main magnetic field. Images were displayed inside the CMR scanner room using an interventional in-room console with a RF-shielded screen (Siemens Healthcare Sector, Erlangen, Germany) to provide real-time visualization for the operator during subsequent device movement and valve deployment.

The delivery devices were advanced at approximately 2 cm/s under real-time TrueFISP imaging. After approximately 20 cm longitudinal device movement, the devices were stopped and the release mechanism applied. In the case of the balloon-expandable stent-valve, the balloon catheter was advanced separately without the guiding catheter to evaluate balloon inflation and deflation. Subsequently, TrueFISP imaging was used for precise real-time monitoring of valve deployment in a plastic tube, which was added to the phantom model to simulate the aortic annulus and ascending aorta. Since the guiding catheter of the balloon-expandable Edwards SAPIEN™ stent-valve revealed severe susceptibility artifacts, the over-the-wire balloon catheter was also used without the guiding catheter to deploy the balloon-mounted valve.

After deployment within the plastic tube, the bioprostheses were again examined with high-resolution T1-weighted 3D fast low-angle shot (FLASH) sequences (see parameters above) to evaluate correct positioning.

### Flow Measurements

Finally, measurements of flow dynamics were performed in a flow phantom model using flow-sensitive phase-contrast (PC) sequences. The custom-built flow phantom consisted of a system of plastic tubes contained in a water-filled barrel and was placed longitudinally along the axis of the main magnetic field as described above for the Plexiglas phantom. The plastic tube system was comprised of three different tubes, namely a cone-shaped inflow part, a connecting piece, and an outflow part with a proximal neck of 23 mm inner diameter to accommodate the stent-valves.

A flow pump consisting of a CMR-compatible piston pump capable of flow rates between 0.1 and 35 ml/s (CompuFlow 1000 MR, Shelley Medical Imaging Technologies, Toronto, Ontario, Canada) and a control unit with proprietary software (SimuFlow III, Shelley Medical Imaging Technologies, Toronto, Ontario, Canada) was used to simulate transaortic in vivo blood flow. Thereto, a physiological, pre-programmed, pulsatile waveform was reshaped with the peak flow rate set to the maximum of 35 ml/s followed by a weaker flow lobe with reversed flow direction (peak reverse flow rate: 15 ml/s) for inducing closure of the valve leaflets. A dedicated ECG trigger output of the flow pump allowed for the compulsory ECG gating of the PC CMR imaging sequences.

PC cine acquisitions were obtained in planes orthogonal to the direction of flow approximately 2 cm downstream and 2 cm upstream from the prosthesis. An ECG-triggered through-plane phase-contrast sequence (2D FLASH) was used (imaging parameters: TR 62 ms, TE 3.5 ms, flip angle 30°, FOV 320 × 220 mm^2^, matrix 192 × 132, bandwidth 555 Hz/pixel, TA 1:52 minutes).

The velocity-encoded value (VENC) of the PC sequence was set to 100 cm/s for these flow phantom experiments. By manually drawing regions of interest over the appropriate areas, PC flow curves were acquired and integrated over time using vendor-provided software (Argus, Siemens Healthcare Sector, Erlangen, Germany).

### Data Analysis

Image quality was evaluated with respect to static visualization of the stent-valves and the delivery devices and with respect to dynamic, real-time visualization during longitudinal motion and during valve deployment. Image quality assessment was performed in consensus by a radiologist and a physicist using the following grading: severe artifacts with poor visualization (1 point), minor artifacts allowing fair visualization (2 points), and excellent visualization with delineation of device details (3 points).

Assessment of RF signal shielding of the inside of the expanded stent-valves due to RF signal caging was expressed as percentage signal loss within the valve lumen on the T_1_-weighted 3D FLASH images with respect to the undisturbed signal in the water/gadolinium solution outside of the stent-valve[22]. For this purpose, signal intensity was measured at regions of interest (ROI) within the stent-valve as well as outside in coronal and axial orientations. To account for inherent spatial signal variations due to different locations of the regions of interest relative to the position of the surface coils, three measurements were taken and averaged. In addition, these T_1_-weighted 3D FLASH sequences were evaluated for regional signal enhancements due to RF resonance effects.

## Results

### High-Resolution CMR

**Figure 4:**
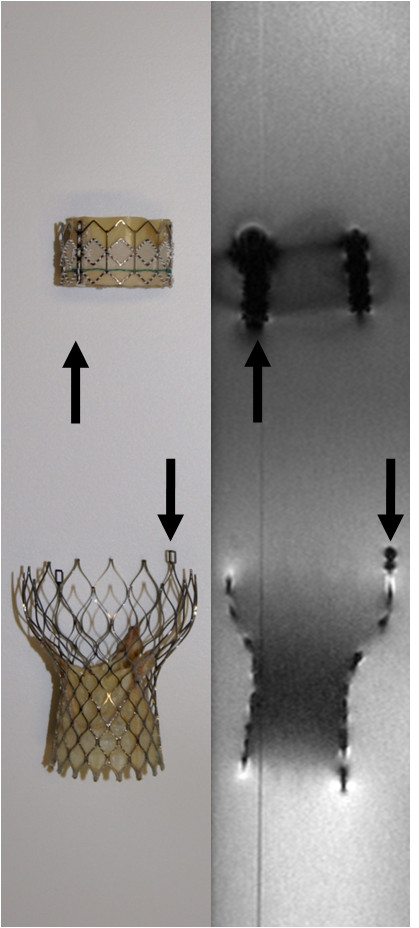
**High-Resolution CMR - Stent-Valves**. Photograph (left side) of the Edwards SAPIEN™ (top) and the Medtronic CoreValve^® ^bioprostheses (bottom) with corresponding single coronal slices from high-resolution T_1_-weigthed 3D FLASH sequences (right side). While RF signal attenuation within the stent was similar for both stent-valves, the Medtronic CoreValve^® ^prosthesis could be depicted in greater detail, even showing the eyelets (arrows) at the outflow tract of the stent. Such a subtle and constrained artifact may potentially be used to indicate orientation before and during valve deployment. This may become relevant in the future of TAVI when rotational orientation with regards to the coronary ostia has to be respected with new devices such as the self-expandable, Acurate RP™ porcine bioprosthesis (Symetis S.A., Lausanne, Switzerland). Likewise, the Edwards SAPIEN™ prosthesis exhibits a somewhat inhomogeneous artifact (arrows) caused by the joint insertion of two adjacent leaflets at the stent-frame, thereby resembling one of the three commissures.

**Figure 5:**
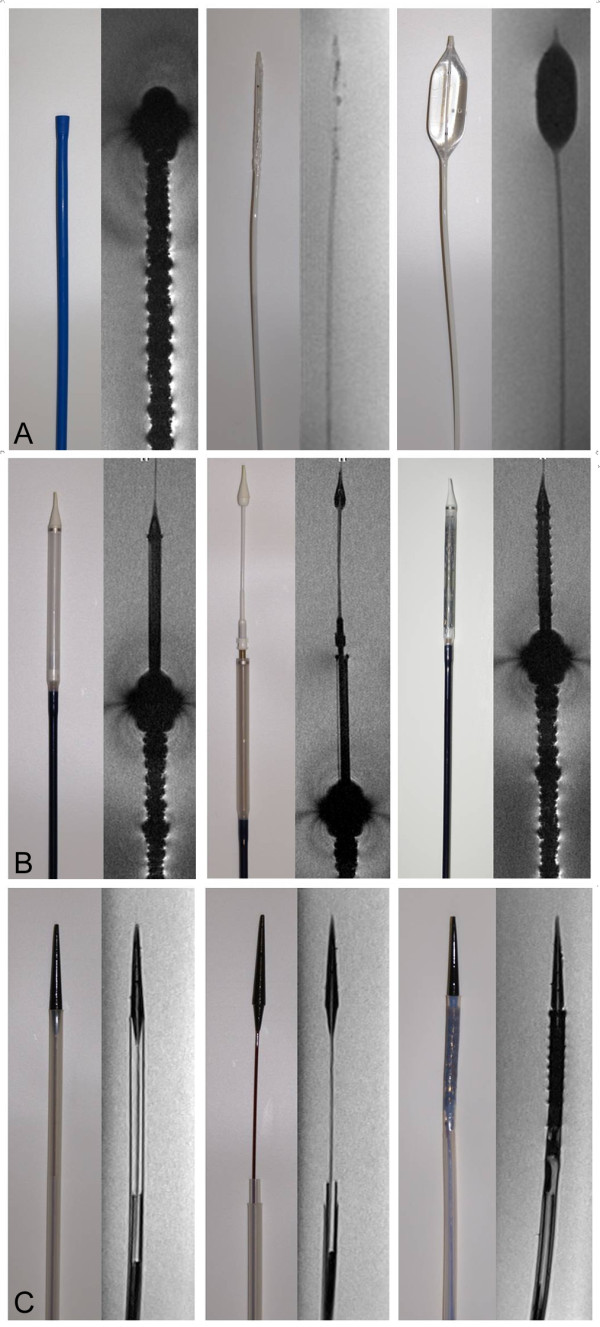
**High-Resolution CMR - Delivery Devices.** a. Left: Photograph of the 24 French RetroFlex delivery catheter for the Edwards SAPIEN™ stent-valve and visualization with high-resolution T_1_-weighted 3D FLASH imaging revealing major susceptibility artifacts caused by metal braiding. Middle: Photograph of the balloon catheter on which the stent-valve is crimped for deployment and corresponding CMR image in static position using real-time TrueFISP imaging. The balloon catheter could be well visualized without any disturbing artifacts. Right: Photograph of the inflated balloon catheter, which could be depicted artifact-free with dark contrast with real-time TrueFISP imaging. b. Left: Photograph of the commercially available delivery catheter of the Medtronic CoreValve^® ^prosthesis and visualization with high-resolution T_1_-weighted 3D FLASH imaging showing severe ferromagnetic artifacts of the catheter shaft but good image quality without disturbing artifacts of the metal-free distal part. Middle: Photograph of the delivery catheter in the release position and corresponding high-resolution CMR image displaying the distal portion of the delivery catheter in great detail. Right: Photograph and corresponding high-resolution CMR image of the loaded delivery catheter. The crimped nitinol stent-valve can be made out easily by its circumscribed ferromagnetic artifacts. c. Left: Photograph of the modified delivery catheter for the Medtronic CoreValve^® ^prosthesis and detailed visualization with high-resolution T_1_-weighted 3D FLASH imaging in excellent quality without any disturbing artifacts. Middle: Photograph of the modified delivery catheter in the release position and corresponding high-resolution CMR image. Right: Photograph and corresponding high-resolution CMR image of the modified delivery catheter with the loaded stent-valve, whose position within the delivery catheter can be easily determined by its circumscribed susceptibility artifacts.

High-resolution T_1_-weighted 3D FLASH imaging provided fair visualization of the tubular, slotted, stainless steel-based balloon-expandable Edwards SAPIEN™ stent-valve with only minor susceptibility artifacts. However, these artifacts rendered the stent slightly larger than its true dimensions, which may be problematic for procedural guidance of TAVI in vivo (Figure 4). In addition, the associated delivery catheter revealed major susceptibility artifacts caused by considerable metal braiding, detrimentally affecting detailed visualization and raising safety concerns in terms of RF-induced device heating (Figure 5a). Moreover, the delivery device also exerted significant ferromagnetic attraction forces in the vicinity of the CMR scanner, which may potentially impose a risk of deflection and migration during TAVI, thus precluding in vivo application. However, the balloon catheter showed excellent visualization without any artifacts when examined separately.

The nitinol-based self-expandable Medtronic CoreValve^® ^prosthesis was excellently visualized, with delineation even of small device details. Its struts and nodes, establishing the repeating diamond cell configuration of the stent frame, could be depicted in great detail. Even the two eyelets at the outflow tract portion of the stent were clearly displayed (Figure 4). While the distal end of the delivery catheter into which the stent-valve is loaded was excellently visualized without any artifacts, the catheter shaft showed severe ferromagnetic susceptibility artifacts similar to the delivery catheter of the Edwards SAPIEN™ prosthesis. Nevertheless, the circumscribed but easily perceived susceptibility artifacts of the crimped nitinol stent still enabled a clear determination of its position within the distal portion of the delivery device, this being an essential prerequisite for precise valve positioning during deployment (Figure 5b).

Overcoming the limitations of the commercial device, the modified delivery catheter for the Medtronic CoreValve^® ^prosthesis, which was designed completely without reinforcement of the shaft by metal braiding, was rendered free of any susceptibility artifacts and provided excellent visualization with great detail and clear determination of the loaded stent-valve position (Figure 5c).

### Real-Time CMR

The rt-TrueFISP sequence allowed for time-resolved display of device motion within the phantom model at a real-time frame rate of 2.5 images/second without any detectable image reconstruction delay or movement artifacts, thus ensuring a sufficiently high temporal resolution for visualization of the dynamic processes of catheter movement and subsequent valve deployment.

**Figure 6:**
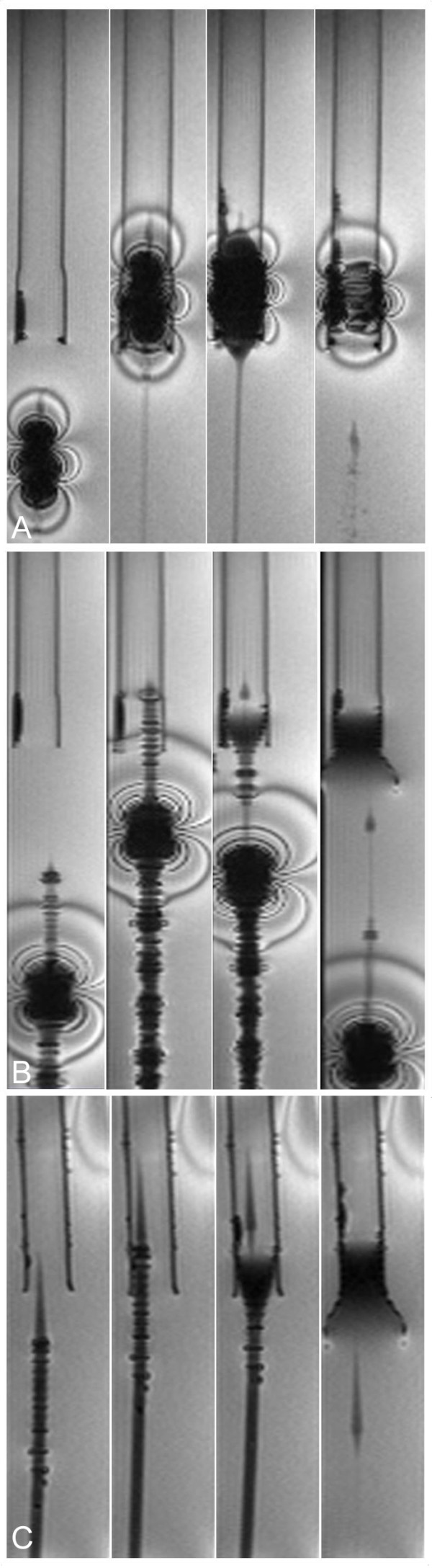
**Real-Time CMR.** a. In vitro deployment of the Edwards SAPIENTM stent-valve crimped onto the balloon catheter: The stent-valve was advanced towards the proximal opening of a plastic tube simulating the aortic annulus (first image). Targeting this "annulus" the valve was positioned (second image) prior deployment by rapid balloon inflation (third image) and deflation. Subsequently, the balloon catheter was pulled back and correct valve position evaluated (fourth image). rt-TrueFISP imaging provided poor visualization with substantial image distortion due to severe ferromagnetic valve artifacts, significantly impairing precise device movement. b. Deployment of the Medtronic CoreValve^® ^prosthesis using the commercially available delivery catheter: The loaded stent-valve was advanced (first image) until the delivery catheter tip was positioned above the "annulus" (second image) before continuously releasing the prosthesis. After expansion of the inflow-part at the targeted position (third image), the valve was fully released and the delivery catheter pulled back, now showing the unsheathed proximal capsule (fourth image). rt-TrueFISP imaging displayed poor visualization of the delivery catheter shaft showing severe ferromagnetic artifacts. In contrast, the distal part covering the crimped stent-valve could be better visualized with less image distortion providing relatively smooth monitoring during stepwise valve release. c. In vitro deployment of the Medtronic CoreValve^® ^prosthesis using the modified, CMR-compatible delivery catheter. The loaded stent-valve was advanced (first image) until the delivery catheter tip was positioned above the simulated annulus (second image). After precise deployment of the inflow-part (third image), the valve was fully released and the delivery catheter pulled back (fourth image). rt-TrueFISP imaging provided excellent, artifact-free device visualization allowing precise imaging guidance. The position of the loaded stent-valve within the delivery device could be clearly discerned by the circumscribed susceptibility artifacts of the crimped nitinol stent, and the position of the retracting delivery catheter sheath could also be clearly followed.

The guiding catheter of the Edwards SAPEN™ stent-valve showed severe susceptibility artifacts exceeding the geometrical constraints of the instrument and rendering the displayed instrument diameter larger than its real diameter. Major image distortion was observed in the device vicinity, as already anticipated from high-resolution imaging, precluding any precise visualization and monitoring of movement in vivo. In contrast, inflation of the balloon catheter alone could be monitored artifact-free with dark contrast of the balloon (Figure 5a). However, the stent-valve mounted on the balloon catheter also showed major susceptibility artifacts in the real-time sequence, potentially hindering exact device placement in vivo (Figure 6a).

Corresponding to the high-resolution CMR images, the distal part of the original delivery catheter for the Medtronic CoreValve^® ^prosthesis could be well visualized without artifacts, while the catheter shaft exhibited severe metal braiding artifacts. However, the distal part could be monitored with good image quality during slow manual release of the stent-valve, ensuring exact CMR-guided placement and deployment of the prosthesis (Figure 6b).

The modified, CMR-compatible delivery device could be rendered completely free of any susceptibility artifacts during real-time imaging. The device constraints were clearly visible to good advantage over the commercially available device. In terms of precise deployment, the modified device showed the same excellent behavior as the commercial device, providing detailed display of the deployment process with sufficiently high temporal resolution (Figure 6c).

### Flow Measurements

**Figure 7:**
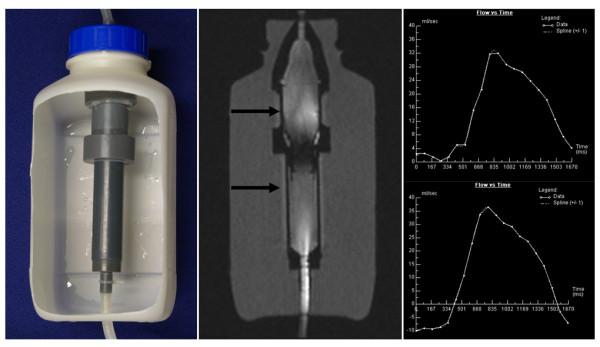
**Flow measurements**. Left: Flow phantom. The custom-built phantom model consists of an interconnected system of plastic tubes contained within a water-filled bottle which is linked in a closed circuit to a CMR-compatible flow pump providing pulsatile flow. Middle: Qualitative assessment of flow exemplified with the Medtronic CoreValve^® ^prosthesis using flow-sensitive phase-contrast cine CMR. Right: Flow curves integrated over time were obtained up and downstream of the stent-valve (arrows) using an ECG-triggered, velocity-encoded, through-plane phase-contrast sequence demonstrating a competent valve without relevant regurgitation.

The flow pump delivered a defined pulsatile waveform with a peak flow rate of 35 ml/s and a peak reverse flow rate of 15 ml/s to simulate transaortic blood flow with ECG triggering for evaluation of flow measurements. Flow dynamics could be representatively assessed both qualitatively and quantitatively for both valves. Assessment of flow could be displayed and measured with ECG-gated cine PC sequences, acquiring 20 phases within the trigger interval. By manually drawing regions of interest over the appropriate flow areas (i.e. ventricular outflow and aortic inflow), PC flow curves could be acquired with reliable determination of flow direction and flow rate, revealing a flow lobe with positive flow direction that was followed by a weaker flow lobe with reversed flow direction. No fundamental differences were observed between the two prostheses. The reverse flow was sufficient to induce closure of the valve leaflets for both valves, thereby revealing or excluding potential aortic regurgitation (Figure 7).

### Data Analysis

Results of the consensus grading of image quality between radiologist and physicist are given in table 1. In general, imaging of the devices under motion was associated with less spatial image resolution and thus reduced image quality compared to static high-resolution imaging. However, this was not necessarily affecting overall image quality grading (Table 1).

**Table 1:** Consensus Grading of Image Quality.

	High-Resolution T1-Weighted 3D FLASH Imaging	Real-Time TrueFISP Imaging
1. Edwards SAPIEN™ Prosthesis and RetroFlex Delivery Device		
- Visualization of the prosthesis	2	1
- Visualization of the delivery device	1	1
- Visualization of the balloon catheter	3	3
- Monitoring of delivery device movement	NA	1
- Monitoring of balloon catheter movement and balloon-inflation	NA	3
- Monitoring of delivery device movement with crimped stent-valve	NA	1
- Monitoring of valve deployment	NA	1
- Visualization of deployed prosthesis	2	1
2. Medtronic CoreValve^® ^and Dedicated Delivery Devive		
- Visualization of the prosthesis	3	3
- Visualization of the delivery device	1 (shaft), 3 (distal part)	1 (shaft), 3 (distal part)
- Monitoring of delivery device movement	NA	1 (shaft), 3 (distal part)
- Monitoring of delivery device movement with loaded stent-valve	NA	1 (shaft), 3 (distal part)
- Monitoring of valve deployment	NA	1 (shaft), 3 (distal part)
- Visualization of deployed prosthesis	3	3
3. Medtronic CoreValve^® ^and Modified, CMR-Compatible Delivery Device		
- Visualization of the prosthesis	3	3
- Visualization of the delivery device	3	3
- Monitoring of delivery device movement	NA	3
- Monitoring of delivery device movement with loaded stent-valve	NA	3
- Monitoring of valve deployment	NA	3
- Visualization of deployed prosthesis	3	3

NA: not applicable

1: severe artifacts with poor visualization; 2: minor artifacts allowing fair visualization; 3: excellent visualization with delineation of device details

The Medtronic CoreValve^® ^prosthesis in combination with the modified delivery device provided excellent visualization during longitudinal device movement and during valve deployment without disturbing artifacts, thereby enabling precise positioning. While the commercial delivery device for the Medtronic CoreValve^® ^prosthesis showed severe artifacts only at the catheter shaft, the entire guiding catheter for the Edwards Sapien™ stent-valve was depicted poorly with severe susceptibility artifacts.

The high-resolution T_1_-weighted 3D FLASH images were used for evaluation of the individual amount of RF shielding within the two stent-valves (Figure 3). For both devices, there was moderate signal attenuation within the lumen of both stent frames, thus precluding direct visualization of the three tenuous leaflets of the integrated pericardial tissue valves in both coronal and axial slices. RF signal attenuation amounted to 59 ± 7% within the balloon-expandable stent-valve and to 57 ± 9% within the self-expandable stent-valve (p = ns).

## Discussion

The present in vitro study evaluated the CMR characteristics of the two currently commercially available TAVI devices, the Edwards SAPIEN™ and the Medtronic CoreValve^® ^bioprosthesis, and of a custom-built delivery device for the Medtronic CoreValve^® ^prosthesis - modified towards CMR safety - in dedicated phantom models as an initial step toward clinical application of real-time CMR-guided TAVI in vivo. Real-time CMR guidance may be desirable for the rapidly emerging TAVI procedure, since it potentially overcomes inherent shortcomings of X-ray fluoroscopy and angiography, which are currently used for imaging guidance. Complementary to mitigation of radiation exposure and nephrotoxicity of contrast media, the TAVI procedure would benefit from real-time CMR guidance due to its inherent unsurpassed soft-tissue contrast and its 2D and 3D imaging capabilities in any oblique orientation during navigation of the large-diameter catheters through the vasculature and, even more importantly, during precise axial positioning of the stent-valve and deployment. So far, only two provocative initial demonstrations in the preclinical era of TAVI have addressed the feasibility and potential value of combined device and tissue imaging by CMR guidance in animal models using entirely hand-made devices far from clinical approval and applicability [23,24]. In contrast, CMR suitability of approved and already commercially available TAVI devices which might be readily transferred into CMR application has not yet been evaluated.

However, collection of precise three dimensional information about soft-tissue anatomy and precise visualization of catheter instruments in relation to the surrounding anatomy has proven to be difficult[3]. The optimal CMR technique to visualize intravascular instruments would be characterized by a high spatial and temporal resolution and, at the same time, a high contrast instrument signature in order to easily identify the instrument in the CMR image[4]. A number of approaches have been developed for depicting vascular instruments by CMR. Basically, active and passive tracking is used to render catheters conspicuous on CMR. While passive catheters are conspicuous based on their intrinsic material properties, active catheters are conspicuous because they contain embedded electronics that interact with the CMR system, usually small RF receiver coils or antennas incorporated into the tip or distal end of a catheter device that are connected to the receiver ports of the CMR scanner with miniaturized coaxial cables running through the catheter body. However, due to RF energy coupling with the RF transmitter coil, these longitudinal cables might be a source of RF-induced heating, thus rendering active catheter tracking potentially hazardous for the patient[4]. In contrast, passive device tracking by CMR uses material-induced susceptibility artifacts of the interventional instrument for visualization. Passive tracking often requires no hardware or instrument modifications and, thus, appears to be particular promising in terms of potential clinical applications.

TAVI appears particularly attractive for a passive CMR-guided approach, since device delivery here is performed through large-diameter vessels and the delivery devices come with a rather large instrument diameter between 18 and 24 French. Given these preconditions, passive real-time visualization of the delivery device can be performed without the need for additional device modifications as needed for active visualization approaches [3,4]. However, essential prerequisites for performing TAVI procedures under CMR guidance are CMR safety and CMR compatibility of the devices; these considerations generally pose the major limitations to clinical application of interventional CMR. CMR-safe and -compatible devices are engineered to be conspicuous, to preclude magnetic attraction and displacement, to avoid distortion of the image and obscuration of the relevant parts of the field of view as a result of large magnetic susceptibility signal voids of incorporated metal components, and to prevent RF related heating during exposure to the high RF energy inherently required to acquire the CMR images. Therefore, catheter delivery devices for use in CMR should not contain any metal braidings or metal cores, neither ferromagnetic, nor electrically conducting. Such metal cores or braidings - which are often used in off-the-shelf catheters designed for fluoroscopically-guided procedures for material reinforcement and to increase stiffness and torqueability - may potentially lead to magnetic attraction forces and large ferromagnetic susceptibility artifacts that exceed the dimension of the actual device by far, thus disturbing CMR images to large extent. Beyond the problem of potentially generating artifacts and disturbing image quality, such longitudinal electrically conducting metal cores and braidings may act as antennas in the CMR environment, locally increasing the radiofrequency (RF) Specific Absorption Rate (SAR) near such metallic implants. The local electric field can be amplified, especially if the implants are composed of long conducting structures that potentially can couple significantly with the RF energy of the body coil. This might lead to excessive local tissue heating if standing resonating waves are generated[25-27]. These resonance effects, however, occur only when the length of wire-like structures or longitudinal implants exceeds one half of the RF wavelength, i.e. around 26 cm for 1.5 T in human tissue[25]. For implants that are small compared to the RF wavelength (e.g., stents, coils, endografts), RF resonance is considered unlikely. Hence, both aortic stent-valves used in this study showed no relevant RF heating in non-clinical testing and have been approved for CMR. According to the official instructions for use, the Edwards Sapien bioprosthesis may be scanned safely in a static magnetic field of 3 Tesla or less, in a spatial gradient field of 720 Gauss/cm or less and with a maximum whole-body-averaged specific absorption rate of 3.0 W/kg for 15 minutes of scanning. The device produces a maximum temperature increase of 0.5°C at a maximum whole-body-averaged specific absorption rate of 3.0 W/kg for 15 minutes of MRI. Similarly, the Medtronic CoreValve^® ^bioprosthesis may be scanned safely in a static magnetic field of 3 Tesla or less, in a spatial gradient field of 1500 Gauss/cm or less and with a maximum whole-body-averaged specific absorption rate of 2.0 W/kg for 15 minutes of scanning, according to the official instructions for use. During testing the device has been shown to produce a maximum temperature increase of 3.5°C at 64 MHz and 3.6°C at 128 MHz for a whole-body-averaged specific absorption rate of 2 W/kg for 15 minutes of MRI.

Our study showed that the delivery device of the Edwards SAPIEN™ stent-valve revealed major susceptibility artifacts caused by considerable ferromagnetic metal braiding, thus also raising concerns regarding RF-related device heating. Additionally, significant ferromagnetic attraction forces were observed, which potentially may impose a risk of uncontrollable migration during CMR-guided TAVI, thereby precluding in vivo application. The stainless steel-based prosthesis itself was not subject to ferromagnetic attraction, but produced susceptibility artifacts exceeding the valve's dimensions; this might hinder exact placement in vivo. In contrast, the nitinol-based self-expandable Medtronic CoreValve^® ^bioprosthesis was excellently visualized, with delineation of even small device details. While the commercial delivery catheter shaft revealed strong ferromagnetic artifacts due to metal braiding similar to those seen in the device for the Edwards SAPIEN™ valve, the distal part containing the mounted stent-valve could be visualized artifact-free and allowed for precise valve positioning and deployment without image distortion at the region of interest. Since engineering data and initial screening of both commercially available delivery systems for metallic components revealed reinforcement of both devices by metal braiding, design modifications of the mechanically simple delivery device of the self-expandable, nitinol-based Medtronic CoreValve^® ^bioprosthesis toward improved CMR safety and CMR compatibility were performed. The modified device was designed completely without reinforcing the delivery device shaft with metal braiding but with preservation of rigidity and flexibility as the key mechanical device properties for in vivo application. These modifications toward enhancing CMR compatibility and CMR safety resulted in artifact elimination and provided excellent real-time visualization of catheter movement and valve deployment using rt-TrueFISP imaging. Therefore, the Medtronic CoreValve^® ^prosthesis may be suited for real-time CMR-based procedural guidance after effective design modifications.

To achieve high instrument to background contrast in the dynamic phantom experiments, a fast rt-TrueFISP sequence was used for data acquisition[28]. Such sequences, when used with high excitation flip angles, provide high blood signal even without administration of a contrast agent. rt-TrueFISP imaging with Cartesian k-space filling provided a frame rate of 2.5 images per second and proved sufficient for direct and precise control of catheter movement and stepwise valve deployment. No detectable image reconstruction delay and no disturbing movement artifacts were observed.

Finally, with respect to CMR surveillance after valve implantation, our study could demonstrate in phantom experiments that dynamic flow assessment of the implanted aortic stent-valves can be performed non-invasively using flow-sensitive PC imaging, which is already used for flow measurements in the clinical evaluation of valvular heart disease[29,30]. Using flow-sensitive PC imaging, aortic regurgitation after valve implantation can be rapidly detected, which is important in clinical practice since hemodynamical relevant aortic regurgitation after TAVI is associated with an increased in-hospital mortality and may require immediate post-dilation of the prosthesis using a balloon[31].

When moving towards preclinical application in animal experiments, the other steps of the TAVI procedure besides valve implantation need to be also taken into consideration, specifically passage of the stenotic aortic valve and its preparation by prior balloon aortic valvuloplasty. While our experiments showed, that the Nu-Med Z-Med II balloon-catheter is suitable for real-time CMR-guidance and may thus be used for balloon valvuloplasty, CMR-compatible guidewires need to be considered for passage of the aortic valve and for propagation of the delivery catheters. According to the literature, there are currently two groups that work towards development and commercialization of first CMR-compatible guidewires [8,9,32-34]. Such CMR-compatible guidewires are polyetheretherketone-based or built from fiberglass or other non-metallic, reinforced components and are passively visualized by their susceptibility artifacts. Whether mechanical stability and other properties will be equivalent to the commercially used products or at least be practical in an in vivo situation remains to be tested in according animal experiments which we are planning to conduct in a swine model.

As a future perspective, it also needs to be evaluated whether preinterventional evaluation for and procedural guidance during TAVI can be performed exclusively under CMR imaging. The TAVI procedure requires thorough preinterventional screening regarding suitability of the peripheral access vessels, the required valve size and potential shift of calcified plaques towards the coronary ostia. At present, this issue is addressed by multimodality imaging using computed tomography, invasive angiography and transesophageal echocardiography. Whether CMR is suited to obviate the need for multimodality imaging is currently unclear and needs to be further evaluated, especially since calcifications can currently not directly be assessed well by CMR.

### Limitations of the Study

Naturally, our in vitro study has some limitations that need to be acknowledged. First, we used a rather simple straight-tube phantom model for device propagation and valve implantation not exactly resembling the more tortuous in vivo geometry which may result in device bending and a device orientation not parallel to the main magnetic field axis. The need for device bending raises concerns about the mechanical properties of the modified delivery catheter for the Medtronic CoreValve^® ^prosthesis. In this study no additional bench-testing was performed for the modified device but it could be demonstrated that flexibility and rigidity was similar to the original, reinforced device by simple manual bending. The fact, that device orientation in vivo may not be parallel to the main magnetic field axis has some potential relevance regarding image quality and RF shielding measurements. Though our measurements give a clear impression of device visualization and the amount of RF shielding in a controlled in vitro setup, this issue needs to be further addressed in the following animal experiments. Second, we did not perform any RF heating measurements for the commercial delivery devices. However, since ferromagnetic attraction forces as well as degraded imaging quality already precluded in vivo application we did not deem those measurements necessary. For the modified delivery catheter for the Medtronic CoreValve^® ^prosthesis RF heating measurements were also not deemed necessary due to its construction without any metallic components. Third, it needs to be mentioned that meanwhile a new generation of the balloon-expandable Edwards valve, the Edwards SAPIEN™ XT prosthesis, has entered the clinical arena. This latest device iteration features a cobalt-chromium instead of stainless steel stent frame, which may be better CMR-compatible. Unfortunately, this new device was not available to us at the time of the experiments. However, this may not be surprising due to the expeditious evolution of this novel technology. Nevertheless, since the delivery catheter is the main limitation precluding CMR-guided TAVI, the conclusions of the present study would most likely not have been affected as even more metal components are involved in the complex mechanism of the NovaFlex delivery catheter which not only offers steerable deflection but also allows the valve to be loaded onto the balloon catheter within the aorta instead of outside the human body.

## Conclusions

The present in vitro study demonstrates that the commercially available nitinol-based, self-expandable Medtronic CoreValve^® ^bioprosthesis is potentially suited for real-time CMR-guided placement using passive device visualization and rt-TrueFISP imaging after effective design modifications of the delivery device. These observations provide a starting point for pursuing real-time CMR-guided TAVI in vivo.

## List of Abbreviations

CMR: cardiovascular magnetic resonance; FLASH: fast low-angle shot; FOV: field of view; LGE: late gadolinium enhancement; PC: phase-contrast; RF: radiofrequency; ROI: region of interest; rt-TrueFISP: real-time fast imaging with steady-state precession; T: Tesla; TA: acquisition time; TAVI: transarterial aortic valve implantation; TE: echo time; TR: repetition time; VENC: velocity-encoded value

## Competing interests

Philipp Kahlert is supported by an internal research grant from the University Duisburg-Essen (IFORES 10+2). Holger Eggebrecht is a clinical proctor for Edwards Lifesciences Inc. and Medtronic Inc. and has received honoraria payment. Ian McDougall is an employee of Evasc Medical Systems, a division of evYsio Medical Devices ULC, Vancouver, Canada.

Brad Decker is an employee of Evasc Medical Systems, a division of evYsio Medical Devices ULC, Vancouver, Canada. The other authors have no commercial, proprietary or financial interests in any products or companies described in this article. The investigated devices were provided by the companies free of charge.

## Authors' contributions

PK has conceived the study, acquired, analyzed and interpreted the data and drafted the manuscript. HE has made substantial intellectual contribution to conception and design of the study and has revised the manuscript for important intellectual content. BP has made substantial intellectual contribution to conception and design of the study and has revised the manuscript for important intellectual content. OK has made substantial intellectual contribution to conception and design of the study, has acquired and analyzed the data and has revised the manuscript for important intellectual content. IMD has made substantial contributions to the published study by designing the modified delivery catheter used in the experiments. BD has made substantial contributions to the published study by designing the modified delivery catheter used in the experiments as senior catheter engineer of Evasc Medical Systems. RE has made substantial intellectual contribution to conception and design of the study and has revised the manuscript for important intellectual content. MEL has made substantial intellectual contribution to conception and design of the study and has revised the manuscript for important intellectual content. HHQ has conceived the study, acquired, analyzed and interpreted the data and drafted the manuscript. All authors have read and given final approval of the manuscript.
